# 

**Published:** 1931

**Authors:** 


CONTENTS.

| | *Page* |
|---|---|
| The Relation of Gynæcology to the Glands of Internal Secretion. By D. C. Rayner, Ch.M., F.R.C.S. .. .. | 1 |
| Ectopic Pregnancy. By R. S. Statham, M.D., Ch.M., F.C.O.G., and H. L. Shepherd, M.B., Ch.M., M.C.O.G. .. .. .. | 15 |
| Necrosis of the Myocardium. By Herbert Rogers, M.B., Ch.B. | 35 |
| Congenital Heart Disease as seen in Elementary School Children. By C. Bruce Perry, M.D., M.R.C.P... .. .. | 41 |
| The Differential Diagnosis of Primary Iritis in the Early Stages. By E. R. Chambers, F.R.C.S. .. .. .. .. | 51 |
| Reviews of Books .. .. .. .. .. .. .. .. .. | 57 |
| Editorial Notes .. .. .. .. .. .. .. .. .. | 65 |
| Meetings of Societies .. .. .. .. .. .. .. .. | 67 |
| The Medical Library of the University of Bristol .. .. | 70 |
| Local Medical Notes .. .. .. .. .. .. .. .. .. | 73 |

